# Psychometric properties of EURO-D, a geriatric depression scale: a cross-cultural validation study

**DOI:** 10.1186/s12888-015-0390-4

**Published:** 2015-02-05

**Authors:** Mariella Guerra, Cleusa Ferri, Juan Llibre, A Matthew Prina, Martin Prince

**Affiliations:** 1 Institute of Memory, Depression and Disease Risk, Avda Constructores 1230, Lima, 12 Peru; 2 Centre for Global Mental Health, Health Service and Population Research Department, Institute of Psychiatry, Psychology and Neuroscience, King’s College London, London, UK; 3 Peruvian University, Cayetano, Heredia, Lima, Peru; 4 Federal University of Sao Paulo, UNIFESP, Sao Paulo, Brasil; 5 Medical University of Havana, Havana, Cuba; 6 Centre for Global Mental Health, Health Service and Population Research Department, Institute of Psychiatry, Psychology and Neuroscience, King’s College London, London, UK

**Keywords:** Depression, EURO-D scale, Psychometric properties, Old age, Validation

## Abstract

**Background:**

Many of the assessment tools used to study depression among older people are adaptations of instruments developed in other cultural setting. There is a need to validate those instruments in low and middle income countries (LMIC).

**Methods:**

A one-phase cross-sectional survey of people aged [greater than or equal to] 65 years from LMIC. EURO-D was checked for psychometric properties. Calibration with clinical diagnosis was made using ICD-10. Optimal cutpoint was determined. Concurrent validity was assessed measuring correlations with WHODAS 2.0.

**Results:**

17,852 interviews were completed in 13 sites from nine countries. EURO-D constituted a hierarchical scale in most sites. The most commonly endorsed symptom in Latin American sites was depression; in China was sleep disturbance and tearfulness; in India, irritability and fatigue and in Nigeria loss of enjoyment. Two factor structure (affective and motivation) were demonstrated. Measurement invariance was demonstrated among Latin American and Indian sites being less evident in China and Nigeria. At the 4/5 cutpoint, sensitivity for ICD-10 depressive episode was 86% or higher in all sites and specificity exceeded 84% in all Latin America and Chinese sites. Concurrent validity was supported, at least for Latin American and Indian sites.

**Conclusions:**

There is evidence for the cross-cultural validity of the EURO-D scale at Latin American and Indian settings and its potential applicability in comparative epidemiological studies.

## Background

Depression is a common and burdensome psychiatric disorder in older people [1-3]. In Low and Middle Income Countries (LMIC) it is difficult to assess its prevalence because of the lack of culturally adapted and validated assessments.

Clinical diagnostic criteria for depression including DSM-5 [4] and ICD-10 [5] are applied to adults of all ages. These may, however, miss clinically significant episodes among older people who do not meet these specific criteria. Some investigators have suggested a syndrome of depression without sadness, thought to be more common in older adults [6,7], and a depletion syndrome manifested by withdrawal, apathy, and lack of vigour [8,9].

Depression symptom scales have been widely used in population surveys to quantify depression burden as a continuum, or to screen for depression of clinical significance in the first phase of a two phase survey design [10-15]. However, only the Geriatric Depression Scale [10,11] and the EURO-D [12] were developed specifically for use in older people, and evidence for their validity comes mainly from high income countries [16-21] [12,22].

We set out to assess the construct validity of the EURO-D in large population-based survey samples of older people living in Latin America, India, China and Nigeria, aiming to assess whether this scale measures the same construct in low and middle income countries with diverse cultures and languages. Measurement invariance would be supported by similar measurement properties, and a common ‘nomological net’ of proximate identifiers of the depression symptom score.

## Methods

### Setting, design and procedures

Comprehensive, one-phase, catchment area population-based surveys were conducted according to the same standardised protocol by the 10/66 Dementia Research Group. The full 10/66 study protocol has been published elsewhere [23]. Surveys were carried out in thirteen sites from nine countries (Cuba, Dominican Republic, Puerto Rico, Peru, Mexico, Venezuela, China, India and Nigeria). Peru, Mexico, China and India included both urban and rural catchment areas; the Nigerian catchment area was predominately rural, while in the other countries participants were recruited only from urban catchment areas. All assessments were carefully translated and adapted into the relevant local languages. All the EURO-D items are derived from the GMS, which is part of the 10/66 assessment. All aspects of assessment methodology, including translation and adaptation have been reported in detail in a previous publication [24]. In brief, the GMS was translated and back translated into Spanish, Mandarin, Hindi, Tamil and Ibo. Meta-analysis of 26 publications of exploratory factor analysis of the GDS reported ‘strong evidence of language differences in the factor structure of the GDS’, being language strongly confounded by other aspects of culture [25]. Acceptability and conceptual equivalence were assessed and reviewed by local informants. Interviews were carried out in participants’ own homes and lasted on average two to three hours. Interviewers were fully trained on the 10/66 protocol by the local principal investigator (PI) and the local study coordinator (SC). The study protocol and the consent procedures, including the witnessed consent procedure, were approved by the King's College London research ethics committee and in all local countries: 1- Medical Ethics Committee of Peking University the Sixth Hospital (Institute of Mental Health, China); 2- the Memory, Depression Institute and Risk Diseases (IMEDER) Ethics Committee (Peru); 3- Finlay Albarran Medical Faculty of Havana Medical University Ethical Committee (Cuba); 4- Hospital Universitario de Caracas Ethics Committee (Venezuela); 5- Ethics Committee of Nnamdi Azikiwe University Teaching Hospital (Nigeria); 6- Consejo Nacional de Bioética y Salud (CONABIOS, Dominican Republic); 7- Christian Medical College (Vellore) Research Ethics Committee (India); 8- Instituto Nacional de Neurología y Neurocirugía Ethics Committee (Mexico); 9-Nnamdi Azikiwe University Teaching Hospital Nnewi Anambra State Ethics Committee, Nigeria. Participants were recruited on the basis of informed signed or witnessed consent; 9-. Ethics committes approved the witnessed consent procedure. The use of the 10/66 Dementia Research Group dataset was approved by the 10/66 principal investigators.

### Depression assessment

Depression was assessed using the Geriatric Mental State (GMS) [26]. Symptoms are ascertained with respect to the last one month. Internationally, the GMS is the most widely used comprehensive clinical mental health assessment for older people. A computerised diagnostic algorithm, the AGECAT (Automated Geriatric Examination for Computer Assisted Taxonomy), groups symptoms to form patterns recognised by a psychiatrist as illness, and identifies them as syndrome cases [27]. Items are later added together to generate affective disorder diagnoses according to ICD-10, and DSM-IV criteria [26,28]. The reliability and validity of the GMS has been demonstrated for in-patient, out-patient and community samples, and in various languages and cultures including Spanish and Chinese. The validity of the GMS/AGECAT algorithm has been investigated in several studies [29,30].

The EURO-D symptom scale was originally developed to compare symptoms of late-life depression across 11 European countries in the EURODEP Concerted Action Programme [12]. The 12 EURO-D items (depressed mood, pessimism, wishing death, guilt, sleep, interest, irritability, appetite, fatigue, concentration, enjoyment and tearfulness) were all taken from the Geriatric Mental State [31]; each item is scored 0 (symptom not present) or 1 (symptom present), generating a simple ordinal scale with a maximum score of 12. In the EURODEP study, internal consistency of the EURO-D, was moderately high with a Cronbach’s alpha ranging from 0.61 to 0.75. However, Principal Components Analysis generated two factors common to nearly every centre: an affective suffering factor (depression, tearfulness, pessimism and wishing death) and a motivation factor (interest, concentration and enjoyment) [12]. The optimum cut-point for the identification of DSM-IV major depression and GMS/AGECAT depression was > =4. Evidence for internal consistency and construct validity of the EURO-D scale was strengthened following its use in the 10 nation European Survey of Health, Ageing, and Retirement in Europe (SHARE) [32]. It was shown to be a hierarchical scale with similar rank ordering of item calibration values across countries. The previously observed two factor structure fitted well in all countries, with similar factor loadings.

Clinical diagnoses of depressive episode (mild, moderate or severe) were classified according to the International Classification of Disease-10 (ICD-10) as a mood disorder with symptoms of sadness, negative self-regard, loss of interest in life, and disruptions of sleep, appetite, thinking, and energy level for more than two weeks that interfere with daily living [5]. ICD-10 diagnoses were derived from the GMS interview, through the application of a computerised algorithm.

### Concurrent validators

We used three indicators to assess the concurrent validity of the EURO-D:

1. Disability was assessed using the World Health Organization Disability Assessment Schedule 2.0 (WHODAS 2.0) [33]. It has high internal consistency, moderate to good test–retest reliability, and good concurrent validity in many clinical populations with chronic disease. The robust cross-cultural measurement properties of the WHODAS 2.0 have been demonstrated in the 10/66 Dementia Research Group population-based surveys [34]; items formed a unidimensional hierarchical scale in all sites, with a common underlying factor structure.
2. Happiness was assessed through the response to GMS question ‘in general, how happy would you say you are: very happy, fairly happy, not very happy, or not happy at all?
3. Subjective global health was assessed through the response to the introductory WHODAS 2.0 question (not used in the overall disability score) – ‘How do you rate your overall health in the past 30 days?’ Options were very good, good, moderate, bad and very bad.

### Analyses

We used the 10/66 data archive (release 3.0) for all analyses.

EURO-D total scale score distributions were summarised according to their mean, median and interquartile range, after inspecting histograms and box plots. The internal consistency of the scale was assessed in each site using Cronbach’s alpha. For each site, the proportion of participants endorsing each of the 12 items (‘item difficulties’) was reported and ranked from 1 (the most frequently endorsed item) to 12 (the least frequently endorsed item) by site.

Mokken analysis was used to test the extent to which the EURO-D items conformed to hierarchical scaling principles in each site. Mokken scaling involves the application of a non-parametric item response model [35] to measure the hierarchical properties of items in a scale, assessing if the items can be ordered by degree of difficulty, so that any individual who endorses a particular item will also endorse all the items ranked lower in difficulty. Three basic assumptions are required for a monotone homogeneity model (MHM): 1) unidimensionality (one latent variable summarises the variation in the item scores in the questionnaire), 2) local independence (after conditioning on the position on the latent trait, the item scores are statistically independent), and 3) monotonicity (for all items the probability of a positive response increases monotonically with increasing values of the latent trait). These assumptions being met, an individual’s position on the latent trait can conveniently be estimated as the rank of the highest item in the hierarchy that they endorse, or their total number of positive responses [36]. Double monotonicity models (DMM) require in addition that for any value of the latent trait, the probability of a positive response decreases with the difficulty of the item. This means that the order of item difficulties remains invariant over all values of the latent trait and thus, that the item response function curves do not intersect [37,38]. To assess single monotonicity, we estimated Loevinger coefficients for each item (H*i*) and for the whole scale (H), where values between 0.3 and 0.4 suggest weak scalability, values between 0.4 and 0.5 moderate, and values above 0.5 strong scalability. We also tested for violations of monotonicity (using the StataloevH monotonicity command) and non-intersection (using the StataloevH nipmatrix command) between pairs of items (minimum violation 0.03, alpha = 0.05), using overall criteria values as an indication of the likelihood of assumption violation; ≤40 ‘satisfactory’, 40 to 79 ‘questionable violation’, 80 and over ‘strongly suggesting an assumption violation’ [39]. Measurement invariance, with respect to hierarchical scale properties was assessed according to the Spearman (non-parametric) correlation between item difficulty ranks between all pairs of sites.

Principal component analysis (PCA) of EURO-D items was carried out using PASW version 18, and confirmatory factor analysis (CFA) using AMOS version 4.0. For PCA varimax rotation was carried out with an Eigenvalue of one as initial extraction criterion. The cut off used to assume that an item loaded on a given factor was 0.60, with a threshold of 0.50 signifying borderline loading. Given the a priori hypothesis of an underlying two-factor solution [40] we then tested and compared between sites the goodness-of-fit of the two factor solution identified in the European SHARE survey, using confirmatory factor analysis. CFA models contain parameters that are (a) fixed to a certain value, (b) constrained to be equal to other parameters, and (c) free to take on any unknown value [41]. In testing for psychometric invariance across sites, two models were fitted and then compared for goodness-of-fit; one in which the factor loadings are unconstrained, that is estimated separately for all countries, and the second in which they are constrained to be equal across countries, the null hypothesis being that items load to a similar extent on the same latent trait or traits across countries. Markedly superior fit of the first model would challenge the hypothesis of measurement invariance. We assessed goodness-of-fit using Akaike’s Information Criterion (AIC) [40], the Tucker-Lewis Index (TLI) [42] and the Root Mean Square Error of Approximation (RMSEA). The lower the AIC value, the better the fit of the model [42]; for the TLI values near 1.0 indicate good fit and those greater than 0.90 are considered satisfactory [43,44]; for the RMSEA values of less than 0.05 indicate close fit and 0.05 to 0.08 reasonable fit for the model [45]. In the final stage of the analysis, we compared the goodness of fit of the two factor solution derived from the European SHARE study with that of a one factor solution, with loadings constrained across sites.

We assessed the psychometric properties of the EURO-D scale, in each site, running receiver operating characteristic (ROC) curve analyses using ICD-10 depressive episode as the reference criterion, plotting sensitivity against false positive rate (1-sensitivity) and estimated the area under the ROC curve (AUROC) with 95% confidence intervals. To calibrate the EURO-D score against ICD-10 depressive episode diagnosis, we used maximum Youden’s index ((sensitivity + specificity)-1) as the criterion for determining the optimal cut-point in each site. The optimal cutpoint for most sites was then applied to all sites, and the sensitivity, specificity and Youden’s index at that cut-point was reported against ICD-10 depressive episode. It is important to note that the EURO-D scale score and ICD-10 diagnosis were both derived from a single GMS interview, administered by the same research worker, with some overlap in the symptoms ascertained. Therefore, this does not represent an independent validation of the EURO-D scale, but rather an attempt to compare its calibration with ICD-10 clinical diagnosis among sites.

The concurrent validity of the EURO-D scale in each site was assessed by measuring Spearman rank correlations with global self-rated health (an inverse correlation hypothesised), WHODAS 2.0 disability (a positive correlation hypothesised) and happiness (an inverse correlation hypothesised).

## Results and discussion

### Results

#### Sample characteristics

Overall, 17,852 interviews were completed in 13 sites from nine countries. A high response rate was obtained, at least 80% in all sites, and exceeding 90% in several sites. Table 1 summarizes the sample demographic characteristics, by country. Women predominate over men in all sites. Educational levels varied widely between sites, the proportion not completing primary education was higher in sites in India, China and Nigeria in comparison to those in Latin America, and was also generally higher in rural than urban sites.

**Table 1:** **Response proportion, sociodemographic characteristics and EURO-D score distributions by site**

	Cuba n = 2944	Dominican Republic n = 2011	P Rico n = 1918	Peru urban n = 1381	Peru rural n = 552	Venezuela n = 1965	Mexico urban n = 1003	Mexico rural n = 1000	China urban n = 1160	China rural n = 1002	India urban n = 1003	India rural n = 999	Nigeria n = 914
**Response proportion**	94 %	95%	93%	80%	88%	80%	84%	86%	74%	96%	72%	98%	98%
**Age (years)** Mean age	74.8	75.2	76.1	75.0	74.1	72.3	74.4	74.1	73.9	72.4	71.2	72.5	72.6
Missing values	(7)	(0)	(2)	(0)	(0)	(4)	(1)	(0)	(0)	(0)	(2)	(0)	(0)
**Gender**													
Female	1913 (64.9)	1325 (65.9)	1289 (67.2)	888 (64.3)	295 (53.4)	1226 (63.4)	666 (66.4)	602 (60.2)	661 (56.9)	556 (55.4)	571 (57.6)	545 (54.5)	539 (58.9)
Missing values	(0)	(2)	(4)	(0)	(0)	(33)	(0)	(0)	(0)	(0)	(15)	(0)	0
**Marital status**													
Never married	275(9.3)	139 (6.9)	118 (6.1)	145 (10.5)	68 (12.3)	189 (9.8)	63 (6.2)	42 (4.2)	3 (0.2)	22 (2.2)	21 (2.1)	5 (0.5)	41 (4.8)
Currently married	1271(43.2	586 (29.3)	931 (48.5)	784 (57.1)	308 (55.9)	921 (47.9)	470 (46.8)	538 (53.8)	829 (71.4)	585 (58.3)	523 (52.2)	481 (48.1)	581 (68.6)
Widowed	928 (31.6)	806 (40.3)	640 (33.3)	367 (26.7)	157 (28.4)	549 (28.5)	395 (39.3)	371 (37.1)	326 (28.1)	394 (39.3)	426 (42.5)	497 (49.7)	225 (26.5)
Separated/divorce	462 (15.7)	465 (23.3)	228 (11.8)	75 (5.4)	18 (3.2)	261 (13.5)	75 (7.4)	48 (4.8)	2 (0.1)	1 (0.1)	32 (3.1)	16 (1.6)	0 (0.0)
Missing values	8	15	4	10	1	45	0	1	0	0	3	0	67
**Education level**													
Did not complete primary	730 (24.8)	1314 (70.9)	446 (23.1)	127 (9.1)	225 (41.3)	601 (31.2)	581 (57.4)	837 (83.7)	385 (33.1)	693 (69.0)	662 (65.9)	855 (85.5)	678 (74.1)
Missing values	8	19	0	8	8	40	2	0	0	0	2	0	0
**Mean EURO-D score (SD)**	2.1 (2.3)	3.0 (2.6)	1.7 (2.0)	2.6 (2.3)	2.4 (2.0)	2.5 (2.4)	2.6 (2.3)	2.3 (2.2)	0.5 (1.2)	0.2 (0.8)	3.2 (2.5)	3.2 (3.1)	2.5 (3.0)
**Median EURO-D score (25th/75th centile)**	1 (0/3)	2 (1/5)	1 (0/3)	2 (1/4)	2 (1/4)	2 (1/4)	2 (1/4)	2 (0/4)	0 (0/1)	0 (0/0)	3 (1/5)	2 (0/6)	1 (0/4)
**Cronbach’s alpha**	0.77	0.76	0.73	0.71	0.64	0.73	0.72	0.70	0.70	0.74	0.72	0.87	0.87

Histograms of EURO-D score distributions (data not provided) indicated that the modal score in all sites, other than urban India, was zero, indicating no depression symptoms. In all sites the distribution was markedly positively skewed. In rural India, the score distribution was biphasic, with peaks at zero to one and five to seven. Mean scores ranged between 1.7 and 3.2, other than in urban China (0.5) and rural China (0.2). Median scores ranged between 1 and 3, and 75th centiles between 3 and 6, other than in urban China (1) and rural China (0). Relatively high score distributions were seen in the Dominican Republic, and India.

The internal consistency of the EURO-D scale Cronbach’s alpha ranged from 0.64 to 0.87, and exceeded 0.70 in almost all sites.

#### EURO-D hierarchical scaling properties

Loevinger’s H coefficients indicated a weak hierarchical scale in Cuba, Dominican Republic, Puerto Rico and China, a moderate hierarchical scale in India and a strong hierarchical scale in Nigeria (Table 2). In Peru, Venezuela and Mexico, Loevinger’s H coefficient fell just below the threshold to support hierarchality. In none of the countries were any significant violations of monotonicity assumptions noted. There were several statistically significant violations of the more stringent double monotone homogeneity (non-intersection) assumptions, but strong evidence of violation was only seen for a minority of symptoms in certain sites. The pattern of item-specific Loevinger’s H coefficients and non-intersection violations did not suggest that any particular items could be omitted to generate a more effective hierarchical scale across countries.

**Table 2:** **Mokken analysis**

	Cuba	DR	Puerto Rico	Peru	Venezuela	Mexico	China	India	Nigeria
EURO 1	0.56	0.49	0.57	0.43	0.42	0.44	0.44	0.52*	0.43
Depression									
EURO 2	0.38	0.25	0.35	0.27	0.24	0.26	0.31	0.49	0.50
Pessimism									
EURO 3	0.37	0.35	0.30	0.29	0.31	0.27	0.51	0.38	0.65
Wishing death									
EURO 4	0.25	0.25	0.23	0.23	0.17	0.18	0.47	0.22	0.53
Guilt									
EURO 5	0.33	0.33	0.29	0.24	0.31	0.24	0.23	0.46	0.45
Sleep									
EURO 6	0.42	0.33*	0.39	0.28	0.36*	0.32	0.38	0.43	0.59
Interest									
EURO 7	0.18*	0.25*	0.27	0.11*	0.22	0.15	0.31	0.37	0.50
Irritability									
EURO 8	0.29	0.28*	0.20	0.22	0.18*	0.18	0.25	0.43	0.26*
Appetite									
EURO 9	0.33	0.32	0.44	0.22	0.27	0.25	0.30	0.29*	0.43
Fatigue									
EURO 10	0.25	0.18	0.20	0.22	0.27	0.19	0.09	0.30	0.56
Concentration									
EURO 11	0.42	0.33*	0.45	0.30*	0.39	0.37	0.34	0.44	0.71
Enjoyment									
EURO 12	0.39*	0.37	0.42	0.31	0.32*	0.34	0.41	0.44	0.55
Tearfulness									
**Loevinger’s coefficient H**	**0.35**	**0.31**	**0.33**	**0.26**	**0.29**	**0.26**	**0.31**	**0.41**	**0.51**

Item-specific and scale Loevinger’s H coefficients, by country, with violations of monotonicity and non-intersection assumptions.

*p = 0.01 to <0.05.

The proportion of participants in each site endorsing each of the EURO-D symptoms is summarized in Table 3. The symptoms are ranked, within each site, in order of frequency of endorsement. The prevalence of individual symptoms and their rank order were similar across Latin American and Indian sites. The prevalence of all symptoms was strikingly lower in Chinese sites, other than tearfulness, which was commonly endorsed in the rural Chinese site. The rank order of symptoms was also somewhat different from that observed in Latin American and Indian sites. The rank order of symptoms in the Nigerian site was strikingly different from those in all other sites. Thus, depressed mood was the most commonly endorsed symptom in all Latin American sites, and the second or third most endorsed symptom in Indian sites. Sleep disturbance and tearfulness were the other commonly endorsed symptoms in those sites. However, in China depressed mood was the fifth endorsed symptom, while the more commonly endorsed symptoms were sleep disturbance, fatigue and irritability in urban China and tearfulness, loss of concentration and loss of interest in rural China. In Nigeria, depressed mood was the fourth most commonly endorsed item, the most frequently endorsed items being loss of enjoyment, loss of interest and fatigue. There was more communality across sites as regards the least frequently endorsed symptoms, which tended to be guilt, wishing death, and (other than Nigeria) loss of enjoyment. The correlations between pairs of sites in the rank orders of item prevalences are presented in Table 4. Spearman rank correlations generally exceed 0.70 among Latin American sites. While the correlation between rank orders for the two Chinese sites is high and statistically significant (0.69), those with Latin American sites lie generally in the range 0.40 to 0.60. Correlations between the rank order of symptom endorsement in Nigeria and those in other sites are generally close to zero, although those with urban China (0.45) and rural China (0.35) are somewhat higher.

**Table 3:** **Prevalence (%) of EURO-D symptoms, by site and rank order of item difficulties**

	Cuba	DR	Puerto Rico	Peru urban	Peru rural	Venezuela	Mexico urban	Mexico rural	China urban	China rural	India urban	India rural	Nigeria
EURO 1	39.6	50.5	39.7	44.0	45.6	39.5	41.5	40.7	3.6	1.6	44.0	42.9	28.5
Depression	(1)	(1)	(1)	(1)	(1)	(1)	(1)	(1)	(5)	(5)	(2)	(3)	(4)
EURO 2	25.3	23.4	11.1	15.1	11.1	24.6	29.8	28.0	6.3	0.9	33.5	46.3	20.4
Pessimism	(3)	(5)	(6)	(7)	(7)	(6)	(5)	(2)	(4)	(9)	(5)	(2)	(6)
EURO 3	14.0	15.2	7.9	8.8	7.1	9.0	12.3	14.5	1.4	0.6	24.4	16.9	9.4
Wishing death	(7)	(11)	(8)	(11)	(11)	(10)	(9)	(6)	(10)	(11)	(7)	(8)	(12)
EURO 4	3.1	4.5	6.1	9.7	8.8	5.0	9.4	7.3	0.3	0.3	7.5	3.0	12.2
Guilt	(12)	(12)	(10)	(10)	(10)	(12)	(10)	(9)	(12)	(12)	(12)	(12)	(9)
EURO 5	33.1	39.2	22.2	26.5	10.8	35.9	30.2	25.3	10.6	1.0	34.3	41.0	22.6
Sleep	(2)	(3)	(3)	(5)	(8)	(2)	(4)	(3)	(1)	(8)	(4)	(4)	(5)
EURO 6	9.0	18.0	3.6	11.1	8.9	10.8	6.8	7.3	3.5	2.0	9.0	8.3	34.8
Interest	(9)	(9)	(11)	(8)	(9)	(9)	(11)	(9)	(6)	(3)	(10)	(9)	(2)
EURO 7	18.4	20.5	14.3	33.1	33.6	25.9	23.3	24.8	8.6	1.4	48.7	34.7	9.7
Irritability	(5)	(6)	(5)	(3)	(2)	(5)	(6)	(4)	(3)	(7)	(1)	(5)	(11)
EURO 8	8.6	18.9	9.3	10.1	16.1	11.0	15.8	13.3	2.5	0.7	19.7	26.1	17.3
Appetite	(11)	(8)	(7)	(9)	(6)	(8)	(7)	(7)	(8)	(10)	(8)	(6)	(7)
EURO 9	17.6	35.2	20.9	36.0	31.2	31.1	34.3	24.5	9.4	1.6	35.4	61.5	29.2
Fatigue	(6)	(4)	(4)	(2)	(3)	(4)	(2)	(5)	(2)	(5)	(3)	(1)	(3)
EURO 10	10.3	17.0	7.8	23.6	25.9	21.4	15.7	10.7	2.0	6.8	18.3	7.8	12.0
Concentration	(8)	(10)	(9)	(6)	(5)	(7)	(8)	(8)	(9)	(2)	(9)	(10)	(10)
EURO 11	9.0	19.3	2.7	6.8	7.1	8.4	5.4	5.1	2.6	1.8	7.9	6.4	39.4
Enjoyment	(9)	(7)	(12)	(12)	(11)	(11)	(12)	(11)	(7)	(4)	(11)	(11)	(1)
EURO 12	22.7	40.3	33.9	30.5	30.2	34.5	34.0	2.9	1.0	34.9	29.9	25.8	15.2
Tearfulness	(4)	(2)	(2)	(4)	(4)	(3)	(3)	(12)	(11)	(1)	(6)	(7)	(8)

**Table 4:** **Non-parametric correlations between pairs of sites for rank orders of EURO-D item difficulties**

	Cuba	DR	Puerto Rico	Peru urban	Peru rural	Venezuela	Mexico urban	Mexico rural	China urban	China rural	India urban	India rural	Nigeria
Cuba	1.00	0.83**	0.83**	0.72**	0.54	0.87***	0.80**	0.67*	0.56	0.19	0.82**	0.74**	0.05
DR		1.00	0.84**	0.75**	0.64*	0.89***	0.83**	0.42	0.56	0.38	0.73**	0.73**	0.40
Puerto Rico			1.00	0.83**	0.74**	0.93***	0.97***	0.56	0.40	0.09	0.85**	0.77**	−0.07
Peru urban				1.00	0.92**	0.89***	0.87***	0.51	0.53	0.37	0.83**	0.71**	0.06
Peru rural					1.00	0.76**	0.80**	0.43	0.37	0.35	0.75**	0.62*	−0.06
Venezuela						1.00	0.91***	0.56	0.59*	0.33	0.83**	0.77**	0.12
Mexico urban							1.00	0.59*	0.46	0.12	0.83**	0.84**	0.03
Mexico rural								1.00	0.69*	−0.38	0.76**	0.78**	−0.03
China urban									1.000	0.04	0.69*	0.75**	0.43
China rural										1.00	0.03	−0.07	0.35
India urban											1.00	0.87***	−0.08
India rural												1.00	0.18

*p = 0.01 to <0.05.

**p = 0.001 to <0.01.

***p <0.001.

#### Factor structure

Bartlett’s tests of sphericity and Kaiser-Meyer-Olkin Measure of Sampling Adequacy suggested that factor analysis was appropriate and feasible in all countries (Table 5). The principal components factor analysis yielded three factors with eigenvalues over one in most countries, with a two factor solution in Cuba, and a four factor solution in Mexico. The first two factors dominated in all countries (cumulative variance 36.4-45.8%). The third factors contributed between 8.4% and 9.3% of scale variance, with eigenvalues between 1.0 and 1.1. In most countries, the first factor was dominated by loadings of the depression and tearfulness items (seven countries), accompanied by lower level and less consistent loadings from items addressing suicidality (five countries), and sleep, appetite and pessimism (four countries each). The second factor was most commonly dominated by loadings of interest and enjoyment items (eight countries), with occasional lower level loadings of concentration (three countries). In Venezuela the second factor was dominated by depression and tearfulness, and the third by enjoyment and interest, while in Nigeria the pattern was reversed. In both of these countries the first factor was dominated by pessimism and concentration, with guilt and suicidality also loading in Nigeria. In other sites, the third factor was loaded on by a variety of items; guilt, with or without suicidality and irritability (five countries). In China, the third factor was loaded upon by somatic items, sleep, appetite and fatigue.

**Table 5:** **Principal components analysis (eigenvalues greater than one) by country**

Country		1st Factor items loading > =0.60 and 0.50-0.59 (*)	2nd factor items loading > =0.60 and 0.50-0.59 (*)	Number of factors with eigenvalues >1.0	Items loading on other factors > =0.60 and 0.50-0.59 (*)
Cuba	KMO = 0.82	Variance = 29.7	Variance = 10.4	2	
	Bartlett’s p < 0.001	Depression, Tearfulness Pessimism, Suicidality*	Enjoyment, Interest		
			Concentration*, Fatigue*		
Dominican	KMO = 0.80	Variance = 27.9	Variance = 10.7	3	Guilt Suicidality
Republic	Bartlett’s p < 0.001	Depression, Sleep, Fatigue	Enjoyment, Interest		
		Appetite*, Tearfulness*	Pessimism*		
Puerto Rico	KMO = 0.78	Variance = 26.2	Variance = 11.0	3	Guilt, Suicidality
	Bartlett’s p < 0.001	Depression, Tearfulness	Enjoyment, Interest		Irritability*
		Appetite*, Sleep*, Fatigue*	Concentration*		
Peru	KMO = 0.74	Variance = 24.3	Variance = 12.1	3	Irritability
	Bartlett’s p < 0.001	Depression, Tearfulness	Interest		Guilt*
		Suicidality*, Pessimism*, Appetite*	Enjoyment		
Venezuela	KMO = 0.73	Variance = 26.2	Variance = 11.1	3	Enjoyment
	Bartlett’s p < 0.001	Pessimism, Concentration	Depression		Interest
		Fatigue*, Sleep*, Irritability*	Tearfulness		
Mexico	KMO = 0.72	Variance = 24.6	Variance = 11.8	4	Appetite
	Bartlett’s p < 0.001	Depression, Tearfulness	Enjoyment		Guilt, Sleep*, Fatigue*
		Suicidality, Pessimism	Interest		Irritability*, Concentration*
China	KMO = 0.80	Variance = 31.2	Variance = 12.2	3	Sleep, Appetite*
	Bartlett’s p < 0.001	Tearfulness, Suicidality	Enjoyment, Interest		Fatigue*
		Depression	Concentration*		
India	KMO = 0.80	Variance = 32.2	Variance = 13.6	3	Guilt
	Bartlett’s p < 0.001	Depression, Pessimism, Sleep	Enjoyment		
		Tearfulness, Appetite*, Irritability*	Interest		
Nigeria	KMO = 0.84	Variance = 42.4	Variance = 13.1	3	Depression, Tearfulness
	Bartlett’s p < 0.001	Pessimism, Concentration	Enjoyment		Sleep, Irritability*
		Guilt, Suicidality	Interest		
**Pooled**	KMO = 0.80	Variance = 29.5	Variance = 11.4	2	
	Bartlett’s p < 0.001	Depression, Tearfulness,	Enjoyment		
		Pessimism*, Sleep*, Suicidality*, Irritability*	Interest		

*Means: p < 0.001.

Given that the findings from the PCA were broadly consistent with the two factor (affective suffering and motivation) model previously identified and found to fit well across European SHARE study countries, we formally tested the goodness of fit of this factor structure across 10/66 countries, using confirmatory factor analysis (Table 6). This two factor model showed a moderately good fit across sites according to RMSEA (<0.05), although less convincingly so according to TLI (0.77, much lower than 0.90, considered acceptable) (Table 7). The models in which loadings were constrained to be equal across countries, and which were freely estimated in each country varied little in terms of AIC, TLI or RMSEA, suggesting measurement invariance. Variance in factor loadings was reduced for affective suffering items when Nigeria (a clear outlier) was omitted, and the model fit of the two factor solution was clearly improved. When the model fit of the constrained two factor model (omitting Nigeria) was compared with that of a one factor solution (omitting Nigeria), the two factor solution was clearly superior according to all absolute and relative goodness of fit indices.

**Table 6:** **Confirmatory factor analysis for affective and motivation factors**

	Affective suffering factor loading							Motivation factor loading				Factor correlation
Country	Dep	Tear	Suic	Sleep	Guilt	Irrit	Fatigue	Interest	Enjoyment	Pessimism	Conc	
Cuba	1	0.77	0.44	0.48	0.09	0.25	0.40	1	0.99	0.83	0.47	0.55
DR	1	0.91	0.47	0.64	0.12	0.46	0.60	1	1.11	0.56	0.36	0.51
Peru	1	0.87	0.25	0.38	0.22	0.22	0.36	1	0.76	0.38	0.52	0.30
Venezuela	1	0.85	0.23	0.44	0.08	0.30	0.36	1	0.91	0.29	0.47	0.37
Mexico	1	0.84	0.28	0.35	0.14	0.24	0.38	1	0.91	0.55	0.39	0.31
PR	1	0.84	0.24	0.39	0.15	0.31	0.34	1	0.89	1.14	0.89	0.48
India	1	0.82	0.47	0.74	0.08	0.59	0.38	1	0.88	0.42	0.37	0.31
China	1	0.77	0.49	0.54	0.09	0.74	0.58	1	0.76	0.33	0.26	0.48
Nigeria	1	1.09	0.86	1.03	0.91	0.76	0.87	1	1.13	0.53	0.39	0.66
Mean (SD)	-	0.86 (0.10)	0.41 (0.20)	0.55 (0.22)	0.21 (0.27)	0.43 (0.22)	0.47 (0.18)	1	0.93 (0.13)	0.56 (0.27)	0.46 (0.17)	0.44 (0.13)
Mean (SD) omitting Nigeria	-	0.83 (0.05)	0.36 (0.12)	0.50 (0.14)	0.12 (0.05)	0.39 (0.19)	0.43 (0.10)	1	0.90 (0.11)	0.56 (0.29)	0.47 (0.19)	0.41 (0.10)
Constrained model	1	0.83	0.39	0.53	0.12	0.39	0.45	1	0.93	0.50	0.41	0.54
Constrained model omitting Nigeria	1	0.82	0.37	0.50	0.11	0.37	0.42	1	0.90	0.50	0.42	0.53
One factor solution omitting Nigeria	1	0.81	0.48	0.63	0.12	0.46	0.55	0.40	0.35	0.68	0.31	-

**Table 7:** **Confirmatory factor analysis model fit**

	Two factor solution				One factor solution
	Model 1	Model 2	Model 3	Model 4	Model 5
	Unconstrained	Constrained	Unconstrained (excluding Nigeria)	Constrained (excluding Nigeria)	Constrained (excluding Nigeria)
X^2^	7594.7	9148.1	6314.6	7438.6	13760.2
DF	387	459	344	407	422
AIC	8008.7	9418.1	6682.6	7680.6	13972.2
TLI	0.77	0.77	0.79	0.79	0.62
RMSEA	0.03	0.03	0.03	0.03	0.04
Model comparison	2 vs 1		4 vs 3		5 vs 4
X^2^ change	1553.4		1124.0		6321.6
DF change	72		63		15
X^2^ change/DF change	21.6		17.8		421.4

DF = Degrees of freedom.

AIC = Akaike Information Criterion.

TLI = Tucker-Lewis Index.

RMSEA = Root mean Square Error Approximation.

#### Calibration against clinical diagnoses

The calibration of the EURO-D depression against ICD-10 clinical diagnosis is summarized in (Table 8). The Area Under the Receiver Operating Characteristic curve (AUROC) ranged from 0.89 and 1.00. The optimal cutpoint for the EURO-D against the reference criterion of ICD-10 depressive episode (using the criterion of maximizing Youden’s index), was 4/5 (a score of five or more) in all of the Latin American sites, rural China and Nigeria. While a lower cutpoint (3/4) would have been selected in rural India, and a higher cutpoint in urban China (6/7) and urban India (5/6), there was actually little difference between Youden’s index at these cutpoints and at the 4/5 cutpoint that was

**Table 8:** **Psychometric properties of EURO-D depression scale, by site, with respect to clinical criteria**

	ICD-10 depressive episode			
	AUROC ^ 1 ^	Optimal cutpoint ^ 2 ^ (Youden’s index)	Sensitivity at 4/5 cutpoint (%)	Specificity at 4/5 cutpoint (%)
Cuba	0.97 (0.96-0.98)	4/5 0.85	97.2	87.7
DR	0.95 (0.94-0.96)	4/5 0.78	93.5	84.0
Puerto Rico	0.97 (0.96-0.98)	4/5 0.90	97.9	91.6
Peru urban	0.94 (0.93-0.96)	4/5 0.77	92.0	84.5
Peru rural	0.96 (0.94-0.98)	4/5 0.87	100.0	87.0
Venezuela	0.95 (0.94-0.97)	4/5 0.79	94.4	84.7
Mexico urban	0.93 (0.91-0.96)	4/5 0.74	89.4	84.1
Mexico rural	0.94 (0.92-0.97)	4/5 0.76	88.9	87.0
China urban	1.00 (0.99-1.00)	6/7 0.99	100.0	97.8
China rural	1.00 (0.99-1.00)	4/5 0.87	85.7	99.6
India urban	0.95 (0.92-0.98)	5/6 0.74	97.4	74.1
India rural	0.89 (0.86-0.91)	3/4 0.63	91.3	69.5
Nigeria	0.93 (0.87-0.98)	4/5 0.79	100.0	79.3

^1^AUROC - Area Under the Receiver Operating Characteristic curve.

^2^Defined by maximizing Youden’s index.

optimal for other sites. At the 4/5 cutpoint, the sensitivity for ICD-10 depressive episode was 86% or higher in all sites and the specificity exceeded 84% in all Latin American and Chinese sites. However, specificity was lower in urban India (74.1%), rural India (69.5%) and Nigeria (79.3%), indicating a relatively high false positive rate using that cutpoint in those sites.

#### Concurrent validity

As hypothesized, EURO-D scores were positively correlated with WHODAS 2.0 disability scores in all sites (+0.15 to +0.48, P < 0.001), Table 9. EURO-D depression scores were inversely associated with global self-rated health in all sites, but at a much lower level in urban China (−0.10, p = 0.001) and rural China (−0.06, p = 0.06) than in other sites (−0.27 to −0.43, p < 0.001). EURO-D scores were inversely associated with happiness in all sites (−0.17 to −0.49, p < 0.001) other than China urban (−0.05, p = 0.12) and rural (−0.01, p = 0.70), and Nigeria (+0.01, p = 0.68)

**Table 9:** **Construct (concurrent) validity of EURO-D scale**

	Global self-rated health	Disability (WHODAS 2.0)	Happiness
Cuba	−0.36	+0.41	−0.49
DR	−0.43	+0.48	−0.32
Peru urban	−0.45	+0.46	−0.41
Peru rural	−0.20	+0.37	−0.17
Venezuela	−0.42	+0.47	−0.24
Mexico urban	−0.36	+0.33	−0.43
Mexico rural	−0.32	+0.32	−0.42
Puerto Rico	−0.43	+0.41	−0.42
India urban	−0.35	+0.37	−0.30
India rural	−0.27	+0.42	−0.39
China urban	−0.10 (p = 0.001)	+0.42	−0.05 (p = 0.12)
China rural	−0.06 (p = 0.06)	+0.15	−0.01 (p = 0.70)
Nigeria	−0.34	+0.38	+0.01 (p = 0.68)

Note: p < 0.001 unless otherwise specified.

.

### Discussion

The results of these analyses extend the evidence for the cross-cultural validity of the EURO-D scale, at least to Hispanic Latin American and Indian settings. We were able to replicate the two factor structure (‘affective suffering’ and ‘motivation’) previously demonstrated in two studies in continental Europe [12,32]. Measurement invariance (common factor loadings and rank order of item difficulties) was demonstrated among Latin American and Indian sites, but the evidence for this was less compelling for Chinese sites, and measurement properties were quite different in Nigeria. Concurrent validity (hypothesized positive correlations with disability scores, and negative correlations with subjective health ratings and happiness) was strongly supported for the Latin American and Indian sites. However, correlations with subjective health ratings were weak in China, and the hypothesised negative correlations with happiness were absent in China and Nigeria.

We assessed the construct validity of the EURO-D in large, population-based surveys in diverse low and middle income country settings, including both rural and urban catchment areas. We used advanced psychometric techniques – confirmatory factor analysis and item response models, as well as concurrent validity and calibration with clinical diagnosis to evaluate cross-cultural construct validity. Findings are directly comparable with similar analyses conducted in continental Europe [32,46]. The main limitations of this study are that we did not carry out a criterion validation using an independent clinical interview, and we did not assess test-retest, inter-interviewer or inter-rater reliability for the EURO-D scale items.

Findings from this study are most directly comparable with those from the SHARE survey [22] and the EURODEP consortium studies [47], in which the EURO-D was administered to as part of the GMS (EURODEP, nine sites in eight European countries, older adults aged 65 years and over), or as a free-standing scale (SHARE, 11 European countries, older adults aged 50 years and over) in cross-sectional population-based surveys. In EURODEP, the mean EURO-D score ranged from 1.3 to 3.6 among countries, and in SHARE from 1.8 to 3.1, similar to the range observed in our 10/66 studies of 1.7 to 3.2 (excluding the low outlier of China). Cronbach’s alpha ranging from 0.61 to 0.75 in EURODEP, and from 0.62 to 0.78 in SHARE, similar to the range from 0.64 to 0.77 observed in most 10/66 sites. The unusually high internal consistency in rural India and Nigeria (Cronbach’s alpha, 0.87) may suggest a problem with response set bias in those sites. The EURO-D demonstrated stronger hierarchical scaling properties in the European countries included in the SHARE survey [32] than in the 10/66 sites in Latin America and India. Nevertheless, the rank of item difficulties was similar, with depression, sleep disturbance and fatigue being among the most commonly endorsed items (low item difficulty), and guilt and wishing death among the least commonly endorsed (high item difficulty). In Nigeria, EURO-D item responses were strongly hierarchical but with a strikingly different rank order of item difficulties than that observed in the other 10/66 sites and in the European SHARE survey countries. Principal Components Analysis generated similar factor structures (affective suffering and motivation) in the current study as in the EURODEP studies [46], the SHARE surveys [32], and in convenience samples of depressed and older people from the general population in the 10/66 Dementia Research Group pilot studies in Latin America, India and China [24]. The two factor solution derived in the European SHARE study fitted moderately well in our current sample, particularly when the Nigerian site was excluded.

As in the SHARE study, depression and tearfulness consistently loaded on Affective Suffering. However, in contrast to the SHARE study interest and enjoyment rather than enjoyment and pessimism dominated the Motivation factor. The clinical diagnosis of ICD-10 depressive episode in the current study was derived from the same GMS interview, using many of the same items that were used to score the EURO-D, the distinction being that particular combinations of symptoms (which needed to be persistent and pervasive) were required to meet the ICD-10 criteria. As such, the favourable validity coefficients cannot be taken as evidence of criterion validity. Such evidence is available from independent clinical assessments in some of the EURODEP studies [12], a clinical validation of the EURO-D scale in Spain [48] and high sensitivity for the detection of severe depression in the 10/66 Dementia Research Group pilot studies in Latin America, India and China [24]. We were, however, able to calibrate the EURO-D scale score against a ICD-10 clinical diagnosis of depressive episode; the optimal cutpoint was 4/5 in most sites, one point higher than the 3/4 cutpoint identified as optimal in the EURODEP consortium studies [12,46]. Concurrent validity of the EURO-D scale has not been assessed in previous studies. Depression among older people has been previously shown to be strongly associated with disability [49-51] and inversely associated with self-reported global health [12]. Although happiness is undoubtedly more than the absence of depression, recent analyses of population-based survey data from the United Kingdom, Germany and Australia indicate that mental ill health accounts for by far the largest component of the variance in lack of life satisfaction, dominating the effects of physical health, demographic and socioeconomic factors [52]. As such, the failure to observe the predicted inverse correlation with self-reported happiness in China and Nigeria does not support the construct validity of the EURO-D in those settings.

Several factors may have contributed to the discrepant measurement characteristics of the EURO-D in China and particularly Nigeria. In the Chinese sites the prevalence of nearly all depression symptoms was strikingly low. This may have impeded the elucidation of the factor structure and assessment of hierarchality, as well as limiting the variance to be explained in correlation with concurrent validators. In China the once popular and prevalent diagnosis of shenjing shuairuo, a neurasthenia like syndrome comprising weakness, fatigue, concentration problems, headache and other somatic symptoms seems in recent years to have been supplanted as the most common diagnosis in epidemiological surveys and clinical practice by depressive and anxiety disorders [53]. This has led some to allege an inappropriate importation of western nosologies that do not match well with Chinese cultural idioms of expression of psychological distress [53]. An alternative standpoint is that ‘mental health literacy’, judged by recognition and appropriate attribution of vignettes of depression and anxiety, is low in Chinese populations both inside and outside of China [54]. In this context, it is perhaps noteworthy that in our study depression was not a common symptom in either the urban or rural Chinese sites, and the sleep disturbance, fatigue and irritability were the three commonest symptoms in the urban site, and tearfulness, lack of concentration and loss of interest in the rural site. The EURO-D factor structure derived from the Chinese sample is consistent with previous observations from rural Thailand [55] where a high prevalence of fatigue was also observed, and where in addition to affective suffering and motivation, sleep and appetite constituted a separate third factor.

Cultural differences in the experience, attribution and communication of psychological distress might also have mediated some of the observed differences in measurement properties in Nigeria. Brain Fag Syndrome, comprising a tetrad of somatic complaints, cognitive impairments, sleep related complaints, and other somatic impairments was recognised as a West African culture bound syndrome in DSM-IV [56]. While originally recognised among students in the early 1960s, it is likely that this reflects enduring and widespread tendencies for the expression of psychological distress, informed by cultural norms and traditional medicine services. In our study, loss of enjoyment and interest, and fatigue were the most commonly endorsed symptoms in Nigeria; however, the rank orders of sleep disturbance and concentration problems were similar to those in other sites. Site-specific factors, some of which may have been culture related, may also have influenced the interaction between the older respondent and the interviewer, impacting on the assessment, ascertainment and recording of symptoms. In Nigeria, interviewers were local school leavers as opposed to graduates (often health professionals) in other sites, and levels of education and literacy among participants were the lowest of any of the 10/66 survey sites. While training for interviewing using the GMS was carried out using standardized and rigorous procedures in all sites, this may have been a particularly challenging task for the young interviewers in Nigeria. Finally, in both Nigeria and China, suboptimal translations and or cultural adaptions for either the happiness question or the EURO-D may have led to an underestimation of the correlations between these variables.

## Conclusions

In conclusion, more work needs to be done to establish the validity of the EURO-D scale, and by extension the GMS interview, when used across cultures as a tool for assessing depression symptom severity, and generating clinical diagnoses. While its cross-cultural measurement properties are for the most part favourable, the case for measurement invariance with respect to its European origins weakens progressively with increasing cultural distance and disparity in levels of human development. Different questions, asked in different ways, may have served better to elicit symptoms of depressed mood in certain cultures. Ethnographically informed qualitative research might help to identify culture-specific idioms of psychological distress (not captured by depression nosologies), among older adults in China and Nigeria. With globalisation, and progressive economic and human development, it may be that cultures will tend to converge around a western consensus of ‘mental health literacy’. If so, one might hypothesise that, through a cohort effect, cross-cultural challenges may be most evident in the assessment of the mental health of older adults.
